# Brain Abscesses by Nocardia: An Interesting Case

**DOI:** 10.7759/cureus.38911

**Published:** 2023-05-11

**Authors:** Shamsuddin Anwar, Sudeep Acharya, Kumar Thapa, Nawaraj Adhikari, Neville Mobarakai

**Affiliations:** 1 Infectious Diseases, Tufts Medical Center, Boston, USA; 2 Internal Medicine, Staten Island University Hospital, Staten Island, USA; 3 Pulmonary and Critical Care, Staten Island University Hospital, Staten Island, USA; 4 Infectious Diseases, Staten Island University Hospital, Staten Island, USA

**Keywords:** brain abscess, internal medicine, tropical infectious diseases, travel health, nocardia species

## Abstract

The successful management of disseminated *Nocardia* infection is not well described in medical literature. Immunocompetent individuals presenting with complicated and widespread *Nocardia* infection is an uncommon phenomenon. We describe an interesting case of a large *Nocardia* abscess in the brain in an immunocompetent patient that was aspirated. The patient clinically improved and was discharged home on a prolonged course of intravenous antibiotics and close outpatient follow-up. He successfully finished the antibiotic therapy for one year, and repeat imaging studies suggested the resolution of the abscess. With this case, we also intend to do a brief literature analysis about the management of brain abscess caused by *Nocardia* species.

## Introduction

*Nocardia* species are gram-positive opportunistic bacteria causing infections ranging from pneumonia and skin infections to disseminated infections such as brain abscess. These infections pose significant challenge in diagnosis and treatment due to the complexity of the presentation and the prolonged duration of antibiotics for the eradication of infection. Here, we describe an interesting case of brain abscess by *Nocardia* in an immunocompetent patient [1,2].

## Case presentation

**Figure 1:**
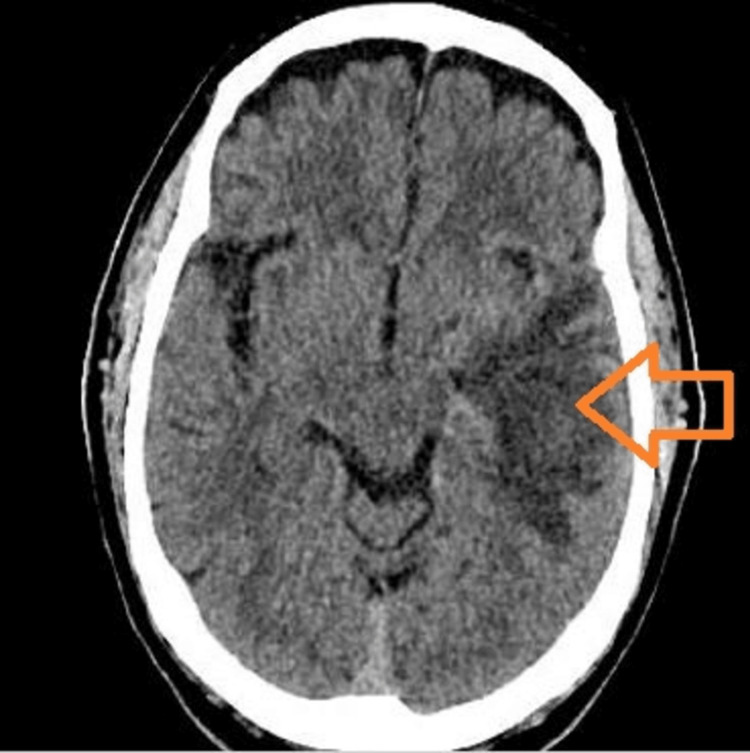
Computed tomography of the head with non-contrast showing left temporal lobe mass with surrounding edema Large left temporal mass (arrowhead) noted on head imaging concerning for malignancy or abscess

A 76-year-old male with a pertinent past medical history of hypertension, dyslipidemia, chronic obstructive pulmonary disease, active smoking (>80 pack-year smoking history), and gout presented to our hospital for persistent headache for a two-week duration accompanied with changes in mentation and rapid memory loss. The patient denied any recent trauma, meningeal signs of infection (fever, chills, night sweats, neck pain, and weight loss), cough, shortness of breath, abdominal pain, or other systemic signs of infection. In the emergency department, the patient was hypertensive (180/80 mmHg), tachypneic (21 breaths/minute), and afebrile with a heart rate of 77 beats/minute. The patient underwent an emergent computed tomography (CT) study of the head without contrast, which demonstrated a left temporal lobe mass of 3 cm with a large amount of surrounding edema, an associated mass effect, and a shift of the third and lateral ventricles to the right of midline suspected to be an intra-axial neoplastic process (primary versus secondary) or infection (Figure 1).

**Figure 2:**
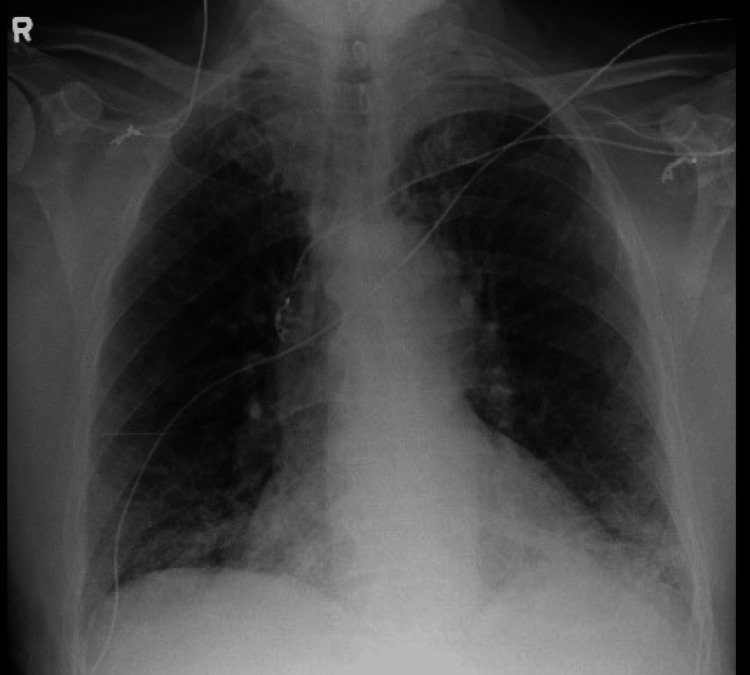
Chest X-ray showing left lung apex with bronchiectasis and scarring

The patient was promptly evaluated by neurosurgery and infectious disease specialists, and workup for suspected malignancy or an intracranial abscess was initiated. He was started on seizure prophylaxis (levetiracetam) and steroids for the cerebral edema and mass effect. No occult malignancy was identified on the imaging studies of the chest, abdomen, and pelvis; however, a new focal opacity at the left lung apex with bronchiectasis favoring focal scarring/sequelae of prior infection was demonstrated (Figure 2).

**Figure 3:**
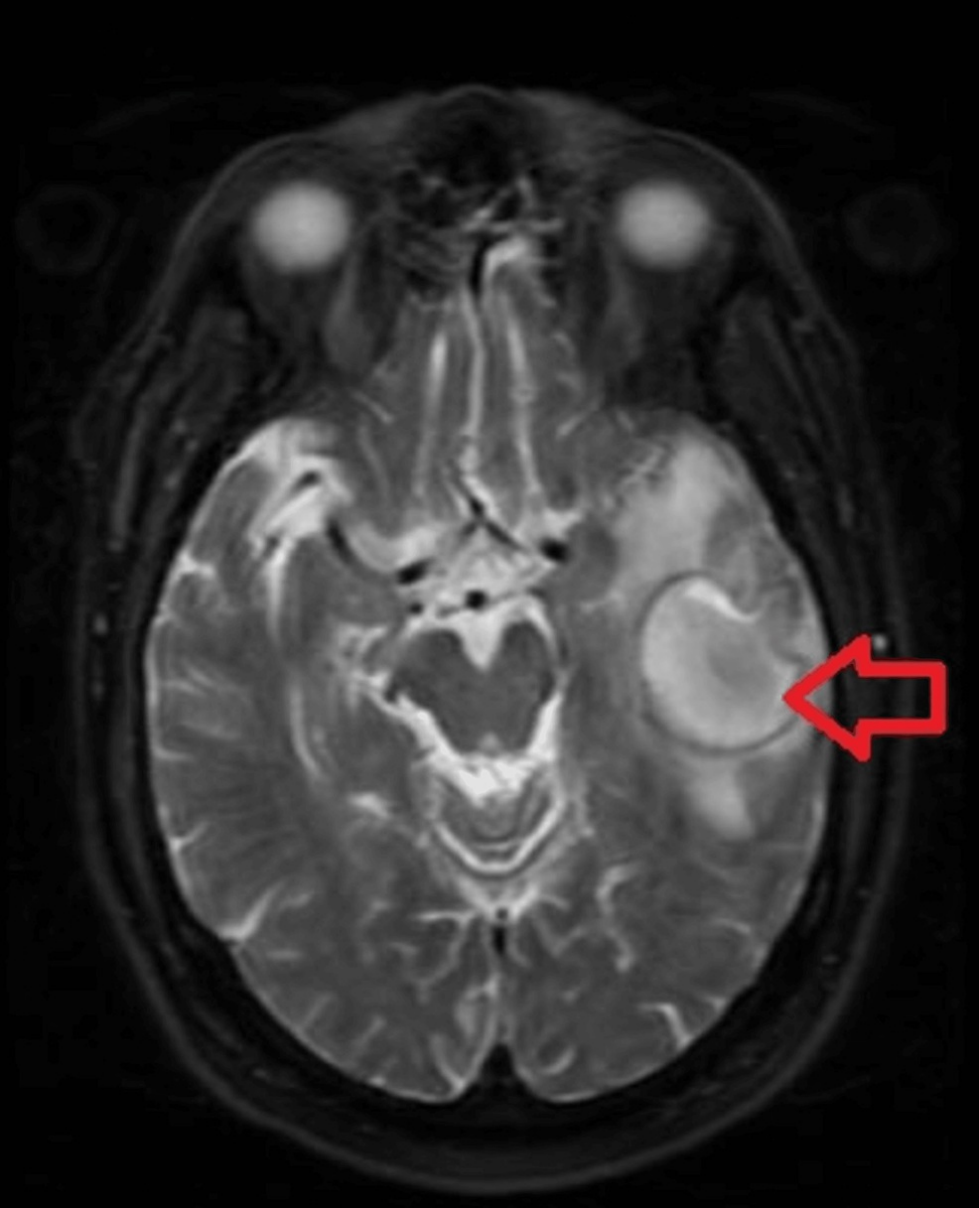
Magnetic resonance imaging of the brain with and without contrast Arrowhead showing left temporal mass consistent with abscess

The patient also underwent magnetic resonance imaging (MRI) of the head with and without contrast, which described a focal mass in the left temporal lobe measuring up to 4 cm with extensive surrounding edema. The signal characteristics on the diffusion-weighted sequence were most compatible with a brain abscess (Figure 3).

The patient underwent burr hole drainage with subsequent aspiration of the intracranial abscess, and cultures were sent. Significant purulence was described and sent for culture, which showed *Nocardia asiatica*, and no fungal contents were noted. He was also tested for HIV and QuantiFERON (Qiagen, Venlo, Netherlands), which were negative. He denied any recent or chronic use of steroids and unpasteurized foods; however, he had a travel history to Puerto Rico, the year prior to presentation, where he had stayed up in the mountains and waterfalls for seven months.

He had routine blood work done after coming back to United States, which had shown a hemoglobin of 19.9 g/dL, monocytes of 1,264, and eosinophils of 589. A routine low-dose CT of the chest one year prior had shown mild bronchial wall thickening with post inflammatory small airway disease and emphysema. During his hospitalization, the blood cultures remained negative, and transthoracic echocardiogram was normal without any vegetations. When the brain abscess cultures showed *Nocardia* species, he was initiated on intravenous trimethoprim-sulfamethoxazole (TMP-SMX), ceftriaxone, and metronidazole. The repeat CT of the head post drainage showed improving left parietotemporal edema with residual postoperative changes. The patient remained in the hospital for a total of 13 days. Since the intravenous TMP-SMX was causing persistent hyperkalemia in the patient, it was switched to intravenous linezolid and meropenem, while ceftriaxone was continued. The patient remained stable throughout the hospital stay and was discharged with peripherally inserted central catheter (PICC) line placement for intravenous antibiotic course.

**Figure 4:**
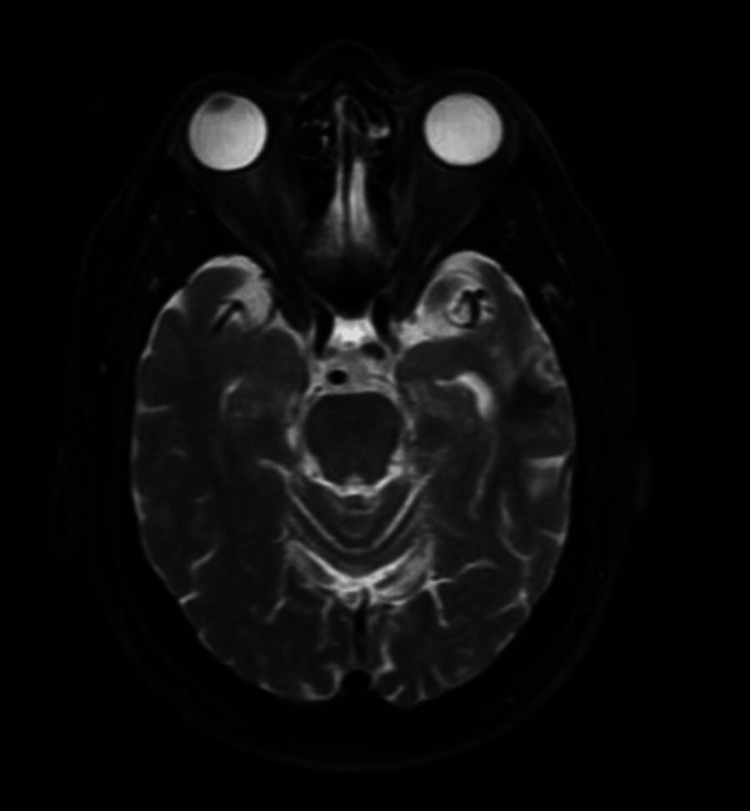
Magnetic resonance imaging of the brain with and without contrast after the completion of antibiotic course (one year)

The susceptibility testing showed *Nocardia* susceptible to imipenem, linezolid, tobramycin, TMP-SMX, cefepime, ceftriaxone, and minocycline. His repeat MRI of the head approximately two months after the diagnosis showed overall decreased size of left temporal lobe abscess and decreased size of surrounding edema. The patient continued to have outpatient follow-up with infectious disease clinic with appropriate tolerance of the antibiotics. He had an MRI of the brain with and without contrast done at the end of the therapy (one year of antibiotic treatment), which showed significantly decreased vasogenic edema with the left temporal lobe, minimal residual gliosis, and no enhancements (Figure 4). The patient is now recommended to follow up with infectious diseases clinic annually.

## Discussion

*Nocardia* species are a diverse group of filamentous gram-positive bacteria that are ubiquitous in the environment. They are commonly found in soil, water (both saltwater and freshwater), and decaying organic matter. These are classically known for opportunistic infections ranging from skin and lymphocutaneous infections to severe diseases involving the pulmonary system and dissemination (brain abscesses) [1,2]. Commonly recognized risk factors for acquiring *Nocardia* infection include immunocompromised states such as HIV, malignancy, renal failure, alcoholism, corticosteroid usage, solid-organ transplant, and chronic lung diseases (such as pulmonary alveolar proteinosis). Although the incidence of *Nocardia* infection is more prominent in immunocompromised states (approximately 62%-65%), it is not unusual to identify cases of *Nocardia* infection in immunocompetent populations such as our patient [3,4].

*Nocardia* species are slow-growing bacteria and can be difficult to culture in a laboratory. Molecular diagnosis utilizing techniques such as matrix-assisted laser desorption ionization-time of flight mass spectrometry (MALD-TOF MS) for 16S ribosomal RNA gene sequencing is now available and a very reliable alternative for the rapid identification of bacteria from the aspirate [5]. The lungs (60%) and skin (21%) are the primary sites of infection in the majority of the cases of nocardiosis. However, other sites such as the bones, heart, kidneys, joints, and brain can also be seen in medical literature [6]. With the evidence of lung finding in the left lung apex of scarring versus previous infection, we suspect that our patient had primary pulmonary nocardiosis with metastatic spread of the infection to the central nervous system. This phenomenon is occasionally described in medical literature [7].

The worldwide incidence of brain abscesses is estimated to be 0.3-1.3 per 100,000 per year, out of which 1% was described to be caused by *Nocardia* [8]. The median age at presentation is noted to be around 64 years; the peak occurrence is between 43 and 75 years of age [4]. Disseminated infection with *Nocardia* particularly in the nervous system can carry high mortality rates (approximately 85%) particularly in immunocompromised states [9]. *Nocardia* brain abscesses are occasionally described in immunocompetent patients, and their mortality rate can be as high as 20% [10-12]. The clinical course of brain abscess with *Nocardia* is typically insidious with symptoms developing over months to years. Fever or other systemic signs of infection may typically be absent as noted in our patient as well except in severe disseminated nocardiosis [13]. The most common symptoms are those of slowly developing cortical mass lesion with headache, seizures, and neurological deficits. Leukocytosis is typically absent, and blood culture and cerebrospinal fluid (CSF) culture are sterile [13,14]. Magnetic resonance imaging (MRI) of the brain is the preferred radiological method to diagnose a suspected nocardial abscess that is described as a ring-enhancing lesion with a necrotic center and surrounding vasogenic edema. They are mostly solitary; however, multiple lesions have also been reported [15]. The most common locations of the abscess are the frontal and parietal lobes, followed by the temporal and occipital lobes. Cerebellar lesion is very rare [16]. In order to differentiate nocardial abscess from intracranial metastatic disease, additional imaging studies such as CT of the chest and abdomen are often warranted. Ultimately, a diagnostic aspiration is required to confirm the diagnosis of abscess formation and further guidance for appropriate antibiotic regimen.

Given the rarity and diverse clinical presentation of disseminated *Nocardia* infection, there is no defined robust therapy, and the choice of antimicrobials is dependent upon available medical literature. As resistance is noted with a variety of isolates of *Nocardia*, treatment plan utilizes two to three agents together in disseminated infections. Trimethoprim-sulfamethoxazole (TMP-SMX) in combination with other antimicrobials is considered the mainstay of therapy against *Nocardia*. It is effective in brain abscess because of its excellent CNS penetration [17]. High parenteral doses (15 mg/kg trimethoprim and 75 mg/kg sulfamethoxazole daily in three to four divided doses) are recommended for at least six weeks, after which the therapy can be reduced or changed depending on the clinical and radiological responses, followed by oral therapy. TMP-SMX is usually combined with one or two antimicrobials such as aminoglycoside, minocycline, imipenem, linezolid, and third-generation cephalosporin. Our patient did not tolerate TMP-SMX due to its side effect, so he was switched to the combination of intravenous meropenem with synergism with ceftriaxone and linezolid [18,19]. The central nervous system nocardiosis should have excision drainage or aspiration of the abscess and then be treated for a total of 12 months and monitored for at least a year further after the completion of treatment as seen in our patient [20].

## Conclusions

Nocardiosis is an uncommon gram-positive bacterial infection commonly seen in immunocompromised hosts. Brain abscesses caused by *Nocardia* not only are difficult to diagnose because of their slow culture growth but also pose unique challenges in the treatment due to the development of resistance and complications. Patients with disseminated infections such as brain abscess require prolonged antibiotic course and close monitoring for complete resolution.
